# Targeting HIV Gag p24 to DICR on dendritic cells induces T cell and potent and long-lasting antibody responses in non-human primates

**DOI:** 10.1186/1742-4690-9-S2-P358

**Published:** 2012-09-13

**Authors:** A Flamar, R Le Grand, V Contreras, S Zurawski, N Dereuddre-Bosquet, I Mangeot, F Martinon, S Oh, J Banchereau, G Zurawski, Y Levy

**Affiliations:** 1 Baylor Institute for Immunology Research, Dallas, TX, USA; 2 CEA, Fontenay-aux-Roses, France; 3 INSERM U955, Faculté de Médecine de Créteil, Creteil, France

## Background

Targeting Dendritic Cells (DCs) with anti-DC receptor antibody-antigen fusion proteins is a novel approach in vaccine development for inducing potent humoral and cellular immune responses.

## Methods

We engineered an anti-human DCIR recombinant antibody cross-reacting with the cynomolgus macaque receptor fused via the heavy chain C-terminus to HIV-1 Gagp24 protein (anti-DCIR.Gagp24). HIV patient PBMC cultures were incubated with anti-DCIR and control hIgG4.Gagp24 fusion proteins. After 10 days, the total T cells were challenged with HIV Gagp24 peptide pools, and then antigen-specific cytokine production was detected using intracellular staining. Macaques were also immunized i.d. 3 times with anti-DCIR.Gagp24 or control hIgG4.Gagp24 with or without polyI:C. Gagp24-specific IgG titers from serum were measured by ELISA and the magnitude of the antigen-specific IFNγ responses was assessed by ELISPOT.

## Results

In vitro, low doses of anti-DCIR Gagp24 prototype vaccine, but not the control hIgG4.Gagp24, expand of Gagp24-specific T cells. These in vitro-expanded antigen-specific T cells were multifunctional, simultaneously producing multiple cytokines (IFNγ, TNFα and MIP-1β). In vivo, in non-adjuvanted naïve animals, serum anti-Gagp24 antibodies were detectable 2 weeks after priming and titers were substantially increased after the 1st and the 2nd boost with anti-DCIR.Gagp24 and were durable, while in the control hIgG4.Gagp24 group the responses were minimal. Poly I:C increased the magnitude of the responses in the anti-DCIR.Gagp24 and hIgG4.Gagp24 groups to a similar level in both groups. T cell responses induced by anti-DCIR Gagp24 could be enhanced after priming with a recombinant modified vaccinia virus Ankara (MVA) encoding HIV Gag/Pol/Nef. Boosting with anti-DCIR.Gagp24 plus poly I:C generated high titers of anti-Gagp24 antibody titers and strongly enhanced IFNγ-producing T cells following priming with MVA HIV Gag/Pol/Nef.

## Conclusion

Our results demonstrate that heterologous prime-boost immunization with vectors and DC-targeting protein-based vaccines is a promising vaccination approach to optimize humoral and cellular immunity for therapeutic and preventive applications against AIDS.

